# Quali-Quantitative Profile of Native Carotenoids in Kumquat from Brazil by HPLC-DAD-APCI/MS

**DOI:** 10.3390/foods8050166

**Published:** 2019-05-16

**Authors:** Helena Maria Pinheiro-Sant’Ana, Pamella Cristine Anunciação, Clarice Silva e Souza, Galdino Xavier de Paula Filho, Andrea Salvo, Giacomo Dugo, Daniele Giuffrida

**Affiliations:** 1 Departamento de Nutrição e Saúde, Universidade Federal de Viçosa, Avenida P.H. Rolfs, s/n, Viçosa 36571-000, Brazil; helena.santana@ufv.br (H.M.P.-S.); nutripamella@gmail.com (P.C.A.); cla_souzabio@yahoo.com.br (C.S.e.S.); 2 Departamento de Educação, Universidade Federal do Amapá, Rodovia Juscelino Kubitschek, Km 02, Jardim Marco Zero, Macapá 68903-419, Brazil; galdinoxpf@gmail.com; 3 Department, of Biomedical and Dental Sciences and Morphofunctional Imaging, University of Messina (Italy), V.le Annunziata, 98168 Messina, Italy; dugog@unime.it (G.D.); dgiuffrida@unime.it (D.G.)

**Keywords:** *Fortunella margarita*, citrus, carotenes, xanthophylls, β-citraurin-laurate, β-cryptoxanthin-laurate

## Abstract

In this study the native carotenoids composition in kumquat (Fortunella margarita) (peel + pulp) from Brazil was determined for the first time by a HPLC-DAD-APCI/MS (high performance liquid chromatography-diode array detector-atmospheric pressure chemical ionization/mass spectrometry), methodology. Eleven carotenoids were successfully identified and quantified in kumquat: four carotenoids in the free form and seven carotenoids in the esterified form. β-citraurin-laurate was the carotenoid found in the highest content (607.33 µg/100 g fresh matter), followed by β-cryptoxanthin-laurate (552.59 µg/100 g). The different esterified forms of β-citraurin and β-cryptoxanthin represented 84.34% of the carotenoids found, which demonstrates the importance of esterification in natural fruits. β-carotene and free xanthophylls (β-cryptoxanthin, lutein and zeaxanthin) represented 5.50% and 14.96%, respectively, of total carotenoids in kumquat. The total carotenoid content of kumquat from Brazil was very high (2185.16 µg/100 g), suggesting that this fruit could contribute significantly to the intake of important bioactive compounds by the population.

## 1. Introduction

The citrus family is one of the first crops in the world, it is estimated that half of the marketed production comes from the Americas and 12% comes from the Mediterranean basin. Citrus cultivation is thought to date back at least 4000 years and is mainly from the Asiatic south-east territories. The estimated global citrus traffic for 2017–2018 was around 6 million tons. The most representative cultures were *Citrus sinensis* (61%), *Citrus reticulata* (22%), *Citrus limon* (11%) and *Citrus paradisi* (6%). In the Americas, the primacy of citrus production lies with Brazil followed by the United States. Sweet oranges are grown in Brazil, mainly in the state of São Paulo, over an area of about 584000 hectares, but also in the Amazonas area of northern Brazil, over about 2.7 hectares. [1].

The *Citrus japonica*, known by the common names of kincan (from Japanese *kinkan*) or cunquate (from Chinese *kumquat*), is a small citrus fruit of the Rutaceae family [2]. It has four major cultivated types, including *Fortunella japonica*, *Fortunella margarita*, *Fortunella crassifolia*, and *Fortunella hindsii* [3].

In eastern countries, this fruit is a part of the regular food habits of the population [4], but in Brazil it is considered exotic, in addition to being little known and commercialized. Among the Brazilian states, São Paulo has the largest production and commercialization of this fruit [5].

Kumquats are native to Central China. They are oval or round fruits with peel and orange smooth. Its flavor varies from acid to sweet. The fruit is rich in vitamins, carotene, pectin, calcium, phosphorus, iron and flavonoids [6]. Kumquats are consumed preferably in natura, whole and in shell. It is also used to make jellies, mousse, jams, marmalades, liqueurs and cachaça [7], preparation of syrups, sauces and also, accompaniment in fruit salads and for landscaping purposes and ornamentation [8,9].

Citrus japonica has been used as a traditional folk medicine in Asian countries to reduce alcohol intoxication and as antidepressants, so they are used either as medicines or as edible fruit [10]. Many studies on antioxidant, antimicrobial and antitumor effects have been carried out on kumquats, however identification of the bioactive compounds in the fruit has received little attention [11].

The most elucidated chemical components in kumquat described in the literature are phenolics compounds and flavonoids. Different phenolic compounds and flavonoids are described in *Fortunella* sp. by HPLC-MS [12,13]. These studies have shown a higher concentration of phenolics in fruit peels, with luteolin and kaempferol being the main flavonoids found in *Fortunella* sp. [8,14,15,16,17].

Studies on carotenoids in kumquat are extremely limited [9,18,19,20]. Agócs et al. [18] studied the qualitative and quantitative composition of carotenoids of kumquat and other citrus species. However, the sample preparation of the carotenoids involved a saponification step, a procedure that does not allow us to evaluate the native composition of the carotenoids.

Studies on the composition of carotenoids in foods are very important because they participate in various biological processes in plants, such as photosynthesis, photomorphogenesis, photoprotection, and development [21]. In animals, provitamin A carotenoids play an essential role in the synthesis of retinol (vitamin A) [22], whereas the xanthophylls lutein and zeaxanthin have been associated in humans with the prevention of age-ralated eye degenerations [23,24].

Carotenoids are molecules made up of a long chain of usually forty carbon atoms; they can by divided into two classes: (a) non oxygenated one named carotenes [25] and (b) oxygenated one named xanthophylls [26]. Moreover, the xanthophylls are usually esterified with fatty acids in nature.

The studies on the content of carotenoids in kumquats concern plants grown in Asia but data are not available for fruits harvested in Brazil [18,19]. Therefore, this work aimed to determine the complete qualitative and quantitative profile of the kumquat carotenoid native composition for fruits collected in the rural area of Viçosa, Minas Gerais, Brazil, through liquid chromatography coupled to the mass detector (HPLC-DAD-APCI-MS).

## 2. Materials and Methods

### 2.1. Chemicals 

The standards of β-carotene, β-cryptoxanthin, lutein, zeaxanthin and physalein, Standard purity was above 98% were purchased from Extrasynthese (Genay, France), and the solvents MeOH (Methanol), MTBE (Methyl-t-butyl ether) and H_2_O (Water) from Sigma-Aldrich (Milan, Italy). 

### 2.2. Collection and Preparation of the Samples 

**Figure 1:**
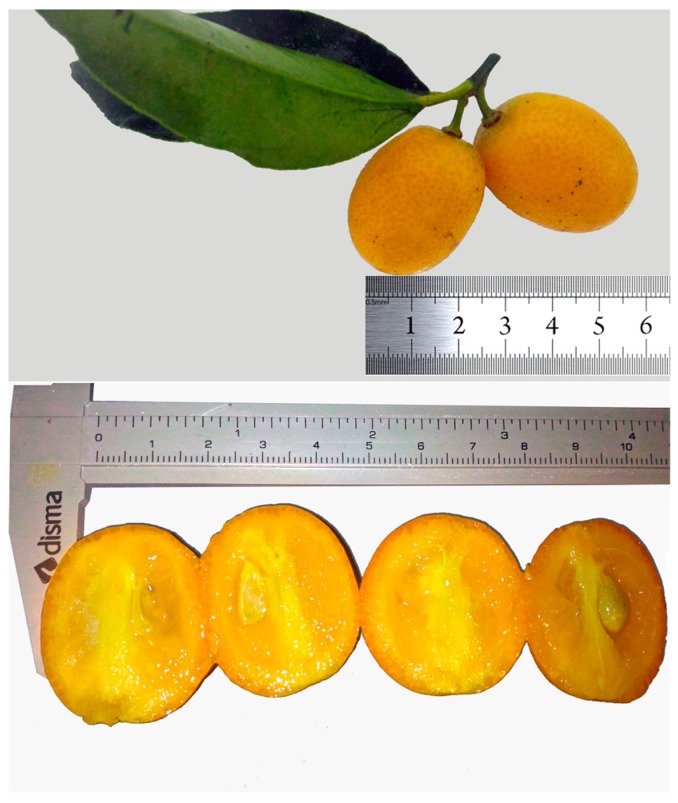
Kumquat (whole fruit and cross-section) from Viçosa, Minas Gerais, Brazil.

The fruits of kumquat (*Fortunella margarita*) (Figure 1) were collected in the morning, in May 2017, in the rural area of Viçosa (latitude 20° 44’ 05’’ S and longitude 42° 51’ 27’’ W), Minas Gerais, Brazil. Samples were collected in four repetitions of approximately 1 kg each. The fruit maturation was determined according to Donadio et al. [27] and defined by the red-orange peel color and the characteristic smell. In addition, ripe fruits were considered as those obtained after their natural fall of the trees or fall after being lightly touched by the hands. 

The species was identified with the help of taxonomists from the Universidade Federal de Viçosa Herbarium through the Angiosperm Phylogeny Group IV [28], where it has already been cataloged and registered in the Virtual Herbarium network with the following records: EAC 48987, HUCO 5197, HPL 8977 and SP 42766.

The samples were transported from the harvest site to the laboratory protected in styrofoam boxes with blocks of ice, within two hours after collection. In the laboratory, the samples were selected for appearance, excluding those with any epidermis injury or mechanical damage due to transport. The fruits were removed from the seeds (peel + pulp) were homogenized in a food processor (RI 7625, Philips, São Paulo Brazil), lyophilized (Liotop-LP510, Liobras, São Carlos, Brazil) and stored in plastic containers with screw caps, covered with aluminum foil stored at −18 ± 1 °C until further analyses. 

### 2.3. Moisture Analysis 

Moisture was determined in triplicate in the oven (SP 200, SP Labor^®^, São Paulo, Brazil), at 65 ± 1 °C, for approximately 72 h [29]. 

### 2.4. Extraction of Carotenoids 

The carotenoid pigments were extracted from the lyophilized material (peel + pulp), according to the recommended procedures by Rodrigues-Amaya et al. [30]. Three grams of the edible portion of the samples (peel + pulp) were crushed by the use of a mortar and pestle, and a few drops of distilled water were added and extracted to color exhaustion with 20 mL of acetone (7 times) in an ultrasonic bath (Labsonic LBS 1-H22.5, Treviglio, Bergamo, Italy) for 10 min each time. Then, the extracts were individually centrifuged (Awel MF20-R, Multifunction Refrigerated Centrifuge, Blain, France) at 4000 rpm, 5 °C, for 10 min in order to withdraw clear solution on the top. The acetonic extracts were pooled together was concentrated to about 25 mL, in a rotary evaporator (Buchi-heating bath B-491, Buchi, Milan, Italy) at temperature below 35 °C. The dry product was diluted with equal volumes (25 mL) of a mixture of ethyl ether and hexane (1:1) and distilled water (50 mL) and worked up with a separating funnel. The lipofilic phase, cleared by the hydrophilic impurities, was evaporated to dryness using a rotary evaporator (Buchi-heating bath B-491 at 35 °C, and the residue was dissolved in 2 mL of MeOH/MTBE (1:1) and filtered in filter units (PTFE, 0.45µm, 13mm, Sigma Aldrich, Milan, Italy) prior to HPLC analysis. Samples were stored at −20 °C until they were analyzed. 

### 2.5. Analysis of Carotenoids by HPLC-DAD-APCI-MS 

The analysis was performed on an HPLC system (Shimadzu, Kyoto, Japan) equipped with a CBM-20A controller, two LC-20AD pumps, a DGU-20A3R deaerator, a SIL-20AC autosampler, a CTO 20AC column oven and an SPD-M20A photo diode array detector. The data were processed with the Labsolution software. For MS analysis a mass spectrometer detector (LCMS-8040) was used, equipped with an APCI (atmospheric pressure chemical ionization) interface, both in positive and negative ionization mode.

The column used was YMC C30 (250 mm × 4.6 mm × 5 μm); the mobile phases: MeOH/MTBE/H2O (81:15:4, solvent A), MeOH/MTBE/H2O (6:90:4, solvent B); the linear gradient used was: 0–100% B from 0 to 140 min. The column temperature was maintained at 30 °C. The flow was 0.8 mL/min and the injection volume was 20 μL.

The UV-Vis spectra were acquired in the range 220–700 nm, while the chromatograms were extracted at 450 nm (sampling frequency: 4.16 Hz, time constant: 0.64 s). The MS was set up as follows: Scan, both APCI positive (+) and negative (-); atomized gas flow (N2): 2.0 L/min; drying gas flow: 5 L/min; Time of the event: 0.06 s; range *m/z*: 300–1200; interface temperature: 350 °C; Desolvation line (DL) temperature: 300 °C; thermal block: 300 °C. The samples were analyzed in triplicate.

### 2.6. Identification and Quantification of Carotenoids

Carotenoids were identified by their UV-Vis spectra, MS spectra, elution order, comparison with the available standard and literature data.

The kumquat carotenoids quantification was performed from the analytical curves. External standards quantitative determination of each compound was performed using all reference materials listed in Section 2.1, in the concentration range from 5 to 50 µg/mL at six concentration levels. The results were obtained from the average of three determinations and the CV% was below 8% in all the LC measurements. The *R* coefficient for the calibration curves was always above 0.9962, with LOD and LOQ values of 0.07 and 0.22 ppm for β-carotene, 0.1 and 0.33 ppm for β-cryptoxanthin, 0.06 and 0.18 ppm for lutein, 0.08 and 0.3 ppm for zeaxanthin, and 0.12 and 0.24 ppm for physalein, respectively.

## 3. Results and Discussion

### 3.1. Carotenoids Qualitative Profile of Brazilian Kumquat 

**Figure 2:**
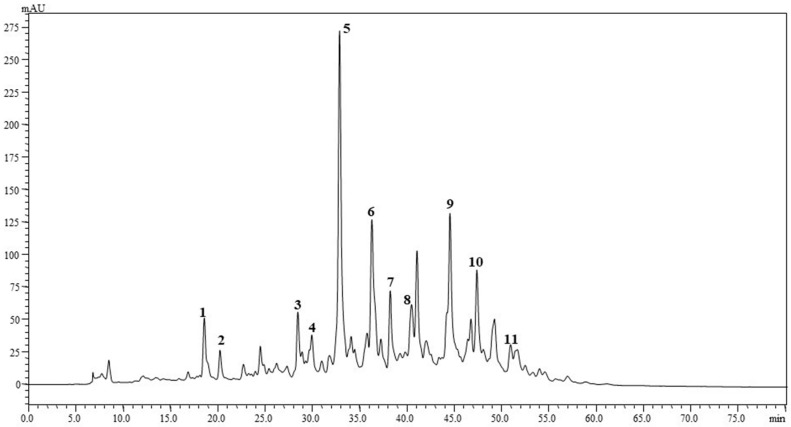
Chromatographic profile of native carotenoids of kumquat from Brazil: peel + pulp. Identification of the compounds are in Table 1.

**Figure 3:**
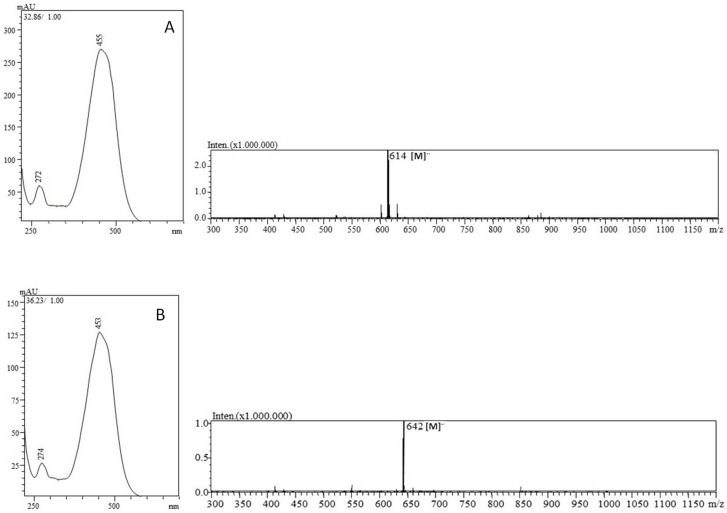
UV-Vis (PDA) and mass spectrum (APCI negative) of β-citraurin-laurate (**A**) and β-citraurin-myristate (**B**) of kumquat from Brazil.

Figure 2 shows the chromatographic profile of the carotenoid composition in not saponified kumquat fruits extracts. The identified compounds are shown in Table 1, together with the UV–Vis and MS spectra information. In Figure 3 are reported the UV-Vis (PDA) and mass spectrum of β-citraurin-laurate and β-citraurin-myristate, detected in the kumquat carotenoid extracts. It can be appreciated that the esterification does not affect the PDA spectra of β-citraurin. 

Moreover, both the molecular ions [M]^−•^ at respectively *m/z* 614 and *m/z* 642 relative to the β-citraurin-laurate and β-citraurin-myristate esters, obtained in the negative APCI mode, are also clearly shown in the same figure. Eleven different carotenoids were identified in kumquat from Brazil; four different β-citraurin esters and three β-cryptoxanthin esters were identified. 

**Figure 4:**
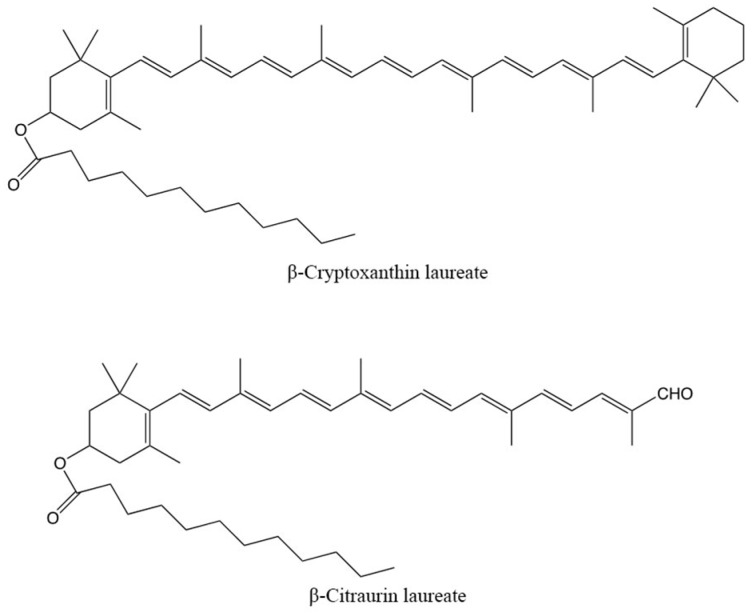
Chemical structures of β-cryptoxanthin-laurate and β-citraurin-laurate, main carotenoids detected in kumquat.

The predominant carotenoids were β-citraurin-laurate and β-cryptoxanthin-laurate, both esterified with lauric acid. The chemical structures of these carotenoids are shown in Figure 4. Interestingly, no free β-citraurin was detected in the present study. 

Shirra et al. [9] detected only 4 carotenoids in kumquat from Italy without saponification (β-carotene, β-cryptoxanthin, lutein and zeaxanthin), which were also detected in the present study. In contrast to our study, Agos et al. [18] reported only β-citraurin and β-cryptoxanthin in the free form in kumquat from Hungary. However, these researchers used saponification after the extraction process and did not quantify the carotenoid esters. Saponification may result in destruction or structural transformation of carotenoids [31]. Huyskens et al. [19] studied the qualitative composition of kumquat carotenoids from Israel by thin-layer chromatography and reported several carotenoids, including β-citraurin, β-cryptoxanthin, lutein, β-carotene and zeaxanthin which were, also determined in the present study. In addition, Huyskens et al. [19] reported that violaxanthin was the predominant component in kumquat from Israel, whereas in our study β-citraurin-laurate was the major component.

Esterification with saturated fatty acids improves the stability of xanthophylls such as β-citraurin and β-cryptoxanthin against heat and UV light, but does not affect their antioxidant activity [32]. During the storage and processing of the fruits the xanthophyll esters were more stable than the free xanthophyll [33]. The pigment β-citraurin is responsible for the citrus reddish color, it derives from of β-cryptoxanthin or zeaxanthin and accumulates in some citrus varieties [34,35]. In these fruits, the accumulation of β-citraurin is not a common event, it is observed only in the flavedos of some varieties during fruit ripening [34]. Frutita, a tropical fruit from Panama, showed a very high content of β-citraurin [36].

Recent studies have shown that β-cryptoxanthin and β-citraurin esterified with lauric acid, myristic acid and palmitic acid are found in the Mandarin Satsuma, [35].

The dietary intake of β-cryptoxanthin has been shown to prevent and reduce some pathologies such as: cancer, diabetes and rheumatism due to its antioxidant activity [37,38,39]. Breithaupt et al. [40] have verified that in chili, papaya, peach and persimmon, β-cryptoxanthin is mainly esterified with saturated fatty acids. Furthermore, the bioavailability of β-cryptoxanthin esters is comparable to the non-esterified form, since fatty acids can be effectively hydrolyzed by β-cryptoxanthin esters before intestinal absorption in the human body [40]. In several citrus varieties, the esterified form β-citraurin is found [35]. As already observed in another study [41], we have found β-cryptoxanthin and β-citraurin in both free and esterified forms.

### 3.2. Carotenoids Quantitative Profile of Brazilian Kumquat 

In the present study, β-citraurin-laurate was the carotenoid found in the highest content in kumquat from Brazil (607.33 µg/100 g fresh matter, representing 27.80% of total carotenoids), followed by β-cryptoxanthin-laurate (552.59 µg/100 g fresh matter, representing 25.31% of total carotenoids). Thus, β-citraurin-laurate and β-cryptoxanthin-laurate represented 53.11% of the total carotenoids found. The different forms of β-citraurin (4 components esterified with fatty acids) and β-cryptoxanthin (1 component in free form and 3 in esterified form) represented 84.34% of the carotenoids found in kumquat from Brazil. Forms of β-citraurin and β-cryptoxanthin esterified with fatty acids accounted for 79.54% of the total carotenoids in kumquat. These results show the importance of the study of the intact carotenoids composition in different matrices (Table 2).

β-carotene was found in smaller amounts (120.19 µg/100 g fresh matter), representing 5.50% of total carotenoids in kumquat, as well as free xanthophylls (β-cryptoxanthin, lutein and zeaxanthin), which represented 14.96% of total carotenoids (326.96 µg/100 g) (Table 2). β-carotene and β-cryptoxanthin possess provitamin A activity, playing a key role in human health [42]. Differently from our results, Wang et al. [20] reported that β-cryptoxanthin was the major carotenoid present in kumquat cultivated in Taiwan, while Schirra et al. [9] found lutein to be the major carotenoid present in kumquat from Italy.

Schirra et al. [9] found a lower concentration, in fresh matter, of β-carotene (33 µg/100 g), β-cryptoxanthin (26 µg/100 g), lutein (44 µg/100 g) and zeaxanthin (24 µg/100 g) in kumquat from Italy when compared to the concentrations of these carotenoids in kumquat from Brazil, reported here for the first time. Wang et al. [41] found contents in dry matter that were much lower than reported here, for lutein (9.9 µg/100 g), zeaxanthin (10.4 µg/100 g), β-cryptoxanthin (183 µg/100 g) and β-carotene (131 µg/100 g) in kumquat cultivated in Taiwan. Carotenoid composition and contents in fruits can be influenced by genetic factors, geographical regions, fruit processing, storage methods Giuffrida et al. [25] and environmental conditions Lu et al. [26], which can explain the differences found in these studies. Besides the climatic factors, the irrigation, soil conditions, fertilizers and herbicides may also affect the carotenoids accumulation [26]. Regarding the carotenoids content in food, Britton [43] have proposed the following classification ranges: low: 0–0.1 mg/100 g; moderate: 0.1–0.5 mg/100 g; high: 0.5-2 mg/100 g; very high: >2 mg/100 g. Thus, the total carotenoid content of kumquat from Brazil was very high (2185.16 µg/100 g).

## 4. Conclusions

In this study the native carotenoids composition in kumquat (*Fortunella margarita*) from Brazil was determined for the first time. Eleven native carotenoids in kumquat from the rural area of Minas Gerais, Brazil were successfully identified and quantified by HPLC-DAD-APCI-MS. Four carotenoids in the free form (β-carotene, β-cryptoxanthin, lutein and zeaxanthin) and 7 carotenoids in the esterified form were identified (β-citraurin-caproate, β-citraurin-laurate, β-citraurin-myristate, β-citraurin-palmitate, β-cryptoxanthin-laurate, β-cryptoxanthin-myristate, β-cryptoxanthin-palmitate). β-citraurin-laurate and β-cryptoxanthin-laurate were the most abundant native carotenoids in kumquat from Brazil. The total carotenoid content of kumquat from Brazil was high (2185.16 µg/100 g), suggesting that this fruit can contribute significantly to the ingestion of important bioactive compounds.

## Figures and Tables

**Table 1:** Compounds identification for Figure 2 (kumquat from Brazil: peel + pulp).

Compound	Identification	Rt (min)	PDA (λnm)	MS (APCI-) *m/z*
1	Lutein	18.5	445, 473	568
2	Zeaxanthin	20.2	449, 476	568
3	β-cryptoxanthin	28.4	428, 451, 478	552
4	β-citraurin-caproate	29.9	454	586
5	β-citraurin-laurate	32.8	455	614
6	β-citraurin-myristate	36.2	453	642
7	β-carotene	38.1	426, 451, 476	536
8	β-citraurin-palmitate	40.4	455	642
9	β-cryptoxanthin-laurate	44.5	428, 450, 478	734
10	β-cryptoxanthin-myristate	47.3	428, 450, 477	762
11	β-cryptoxanthin-palmitate	50.9	428, 451, 478	790

Rt: retention time; PDA: photodiode array; λnm: wavelength of maximum absorption; MS: mass spectrometry; APCI: atmospheric pressure chemical ionization.

**Table 2:** Carotenoid content in kumquat from Brazil (peel + pulp).

Compound	Carotenoid	Content in Lyophilized Kumquat (µg/100 g) *	Content in Fresh Kumquat (µg/100 g) *	Carotenoid Composition (%)
1	Lutein	873.05 ± 93.51	144.35 ± 15.45	6.61
2	Zeaxanthin	469.55 ± 25.52	77.63 ± 4.21	3.55
3	β-Cryptoxanthin	634.93 ± 55.91	104.98 ± 9.25	4.80
4	β-Citraurin-caproate	721.81 ± 20.04	119.34 ± 3.32	5.46
5	β-Citraurin-laurate	3673.28 ± 81.78	607.33 ± 13.63	27.80
6	β-Citraurin-myristate	421.88 ± 9.52	69.75 ± 1.58	3.20
7	β-Carotene	726.96 ± 110.60	120.19 ± 18.29	5.50
8	β-Citraurin-palmitate	801.27 ± 66.35	132.48 ± 10.98	6.06
9	β-Cryptoxanthin-laurate	3342.25 ± 126.10	552.59 ± 20.75	25.31
10	β-Cryptoxanthin-myristate	927.77 ± 82.92	153.39 ± 13.71	7.02
11	β-Cryptoxanthin-palmitate	623.73 ± 46.34	103.13 ± 7.67	4.72
Total carotenes		726.96	120.19	5.50
Total free xanthophylls		1977.53	326.96	14.96
Total esterified xanthophylls		10511.99	1738.01	79.54
Total carotenoids		13,216.48	2185.16	100

* Mean of 3 repetitions ± standard deviation (SD). The carotenoid content in the fresh kumquat was calculated based on the average moisture content (*n* = 3) of the lyophilized fruit (14.08%) and the fresh fruit (81.16%).
